# A correlation study between virulence factors and multidrug resistance among clinical isolates of *Proteus mirabilis*

**DOI:** 10.1007/s42770-023-01080-5

**Published:** 2023-08-03

**Authors:** Mai Elhoshi, Eglal El-Sherbiny, Amel Elsheredy, Aliaa Gamaleldin Aboulela

**Affiliations:** Department of Microbiology, Medical Research Institute, Alexandria University, Alexandria, Egypt

**Keywords:** Multidrug resistant *P. mirabilis*, *P. mirabilis* virulence factors, Biofilm formation in *P. mirabilis*, *P. mirabilis* virulence genes

## Abstract

Treatment of *Proteus mirabilis* infections is a challenge due to the high abundance of virulence factors and the high intrinsic resistance to antimicrobials. Multidrug resistance (MDR) and extensive drug resistance (XDR) further challenge the control of *P. mirabilis* infection. This study aimed to investigate the correlation between virulence determinants and multidrug resistance in 100 clinical isolates of *P. mirabilis* collected in Alexandria from December 2019 to June 2021. Susceptibility to antimicrobials was tested by the Kirby Bauer method. Detection of swarming, urease, protease, hemolysin, and biofilm formation was performed phenotypically and by PCR amplification of *zap*A, *fla*A, *ure*C, *mrp*A, *atf*A, *uca*A, *hpm*A, and *lux*S. MDR and XDR were detected in 34% and 5%, respectively. All isolates were positive for motility, swarming, urease, and protease production. Ninety percent were positive for hemolysin production, while 73% formed biofilm. All isolates possessed the *ure*C and *zap*A genes. The *lux*S, *fla*A, *uca*A, *hpm*A, *mrp*A, and *atf*A genes were detected in 99%, 98%, 96% 90%, 89%, and 84%, respectively. The presence of a single biofilm-related gene was statistically correlated with non-biofilm production (*P*= 0.018). It was concluded that *P. mirabilis* isolates from catheterized-urine samples were significantly associated with biofilm formation. MDR and virulence were not statistically correlated. A significant positive correlation was detected between some virulence genes in *P. mirabilis*. Non-MDR isolates of *P. mirabilis* had a high abundance of virulence factors with no statistically significant difference from MDR. Most of the MDR and all XDR isolates could produce biofilm.

**Supplementary Information:**

The online version contains supplementary material available at 10.1007/s42770-023-01080-5.

## Introduction

*Proteus* spp. belong to the order *Enterobacterales* and to the family *Morganellaceae* [1]. Clinically important *Proteus* spp. include *P. mirabilis*, *P. vulgaris*, and *P. penneri* [2]*. P. mirabilis* is a common causative agent of a diversity of clinical infections such as urinary tract infections (UTI), wound and burn infections, prostatitis, meningitis, otitis media, and rarely respiratory tract infections [3].

Treatment of *P. mirabilis* infections is a challenge due to intrinsic and acquired antimicrobial resistance. *P. mirabilis* is characterized by its intrinsic resistance to many antimicrobial agents: colistin, polymyxin, nitrofurans, tigecycline, and tetracycline [4].

With the extensive and unrestricted use of antimicrobial agents, acquired multidrug resistance (MDR) and extensive drug resistance (XDR) have been commonly encountered among clinical isolates of *P. mirabilis*, posing marked challenges to the control of infection by this bacterial species [5].

The high abundance of virulence factors in *Proteus* spp*.* further augments its impact as a potential threat to public health. *P. mirabilis* possesses a variety of virulence determinants such as fimbriae, flagellae, urease enzyme, hemolysin production, protease enzyme production, biofilm production, and quorum sensing [6].

Fimbriae are responsible for adherence to uroepithelial cells or medical devices, causing urinary tract infection [6] as well as adhesion to wound extracellular matrix proteins such as collagen and fibronectin causing wound infections. The most common fimbriae are mannose-resistant *Proteus-*like fimbriae (MR/P), uroepithelial cell adhesion (UCA/NAF), ambient temperature fimbriae (ATF), *P. mirabilis* fimbriae (PMF), and *P. mirabilis* P-like fimbria (PMF) [7]. Flagellae are utilized for spread to other sites as the migration to the upper urinary tract causing pyelonephritis and the dispersal of biofilm from catheters to the urinary tract [8, 9].

Urease enzymes hydrolyze urea into carbon dioxide and ammonia, which renders the pH of the environment alkaline [10]. In UTI, the alkaline pH causes the precipitation of polyvalent cations such as calcium and magnesium resulting in stone formation [11]. In wound infections, the alkaline pH contributes to delayed wound healing [12].

Hemolysins are secreted toxins produced by *P. mirabilis*, which insert into host cell membranes, causing pore formation, and cytotoxicity, hence facilitating the invasion [13]. In addition, *P. mirabilis* produces ZapA metalloproteases which protect the organism from the host defense by cleaving immunoglobulins, IgA, and IgG [8].

*P. mirabilis* forms biofilms on chronic wound infections [14] and in urinary tract infections especially on catheters [6]. *P. mirabilis* has a unique ability to form biofilms of crystalline nature, owing to the urease activity. This leads to encrustation and obstruction in many cases [8].

Quorum sensing is the main regulator of many virulence factors. It is of particular importance in regulating the multicellular and coordinated processes of swarming and biofilm formation. Quorum sensing in *P. mirabilis* involves autoinducer-1 which is controlled by *lux*R genes and autoinducer-2 which is controlled by *luxS* gene [15].

This study aimed to investigate the correlation between virulence determinants and multidrug resistance in clinical isolates of *P. mirabilis.*

## Materials and methods

### Sample collection

The study was performed on 100 isolates of *P. mirabilis*, collected from clinical samples submitted at the Microbiology laboratory of the Medical Research Institute, Alexandria University. Isolates were collected along the period from December 2019 to June 2021. The isolates were collected from mid-stream urine samples, catheter-collected urine, and wound swabs.

### Identification of isolates

Colonies were presumptively identified as *Proteus* species by observing the formation of swarming on blood agar and the growth of smooth, non-lactose fermenting colonies on MacConkey agar after incubation at 37°C under aerobic conditions for 16–24 h. Standard biochemical tests were used for further species identification of *P. mirabilis*. The detection of *ure*C gene by PCR was employed for further genotypic confirmation of *P. mirabilis* species identification, as previously described [16].

### Antimicrobial susceptibility testing

Kirby Bauer disk diffusion method for susceptibility testing of the isolates was performed, and the results were interpreted as per the CLSI 2021 recommendations [17]. Nineteen antibiotic disks (Oxoid, UK) were tested: ampicillin (10 μg), amoxicillin-clavulanate (20/10 μg), ampicillin-sulbactam (10/10 μg), piperacillin-tazobactam (100/10 μg), cefepime (30 μg), cefotaxime (30 μg), ceftriaxone (30 μg), ceftazidime (30 μg), aztreonam (30 μg), ertapenem (10 μg), imipenem (10 μg), meropenem (10 μg), gentamicin (10 μg), tobramycin (10 μg), amikacin (30 μg), ciprofloxacin (5 μg), levofloxacin (5 μg), ofloxacin (5 μg), trimethoprim-sulfamethoxazole (1.25/23.75 μg). Acquired resistance to antibiotics (at least one) belonging to three or more categories of antimicrobial agents was described as multidrug resistance (MDR). Isolates sensitive to only one or two categories of antimicrobials are classified as extensive drug-resistant (XDR) isolates [18].

### Detection of virulence factors of *P. mirabilis*

#### Phenotypic detection of virulence factors

##### Urease production

The isolates were cultured on urea agar medium by stab. The tubes were incubated at a temperature of 37°C for 24 h. The change of color from yellow to magenta was a sign of a positive result [19].

##### Protease production

Skim milk agar (Himedia) was inoculated with the test isolates and incubated at 37°C for 24 h. A positive reaction appeared in the form of a clear zone that developed around the colonies [20].

##### Hemolysin production

Tube hemolysis assay was performed by inoculating 2 mL of nutrient broth with bacteria. One hundred microliters of 1% washed human red blood cells (RBCs) was added, and the media were incubated for 24 h at 37 °C. Non-hemolyzed red blood cells settled to the bottom of the test tube and formed a button. No button was observed if the cells were lysed by hemolysin [21].

##### Formation of biofilm

The microtiter plate (MTP) assay was performed to test the isolates for biofilm formation. Briefly, a bacterial suspension was prepared in MHB supplemented with 1% glucose. Following the adjustment of the suspension to 5×10^7^ CFU/mL, 200 μL was used to inoculate the wells of a 96-well MTP. After overnight incubation at 37°C, the content of the wells was discarded, and the wells were washed with normal saline. Methanol (99%) was added to the biofilms for fixation. The biofilms were then stained with 1% crystal violet for 20 min. The plate was washed with normal saline to get rid of excess dye. Finally, 200 μL of ethanol (99%) was added to release the bound crystal violet. The optical density (OD) of each well was measured at 620 nm using an MTP reader. The strength of biofilm formation was calculated in relation to the OD of negative control wells [22].

#### Genotypic detection of virulence factors

##### DNA extraction

Genomic DNA was extracted from *P. mirabilis* isolates by boiling method as previously described. In brief, several colonies from a fresh overnight culture of the isolates were washed with Tris-EDTA (TE) buffer and the pelleted cells were resuspended in 250 μL TE buffer by vortexing. The bacterial suspension was incubated in a boiling water bath for 10–15 min, immediately chilled on ice for 2 min, then centrifuged at 14000 rpm for 15 min. The supernatant was transferred into a new tube and was used as the stock DNA extract, which was 10 folds diluted and used as a template for PCR [23].

##### Amplification of virulence genes by multiplex PCR

Conventional PCR was used for the amplification of 8 virulence genes using specific primers: *zap*A [24] encoding extracellular metalloprotease, *fla*A [25] for flagellae, *ure*C [26] for urease enzyme large subunit, *mrp*A [27] for mannose-resistant *Proteus-*like fimbria, *atf*A [28] for ambient-temperature fimbriae, *uca*A [29] for uroepithelial cell adhesin fimbriae, *hpm*A [25] for hemolysin, *lux*S [15] for quorum sensing.

Six genes were detected in 3 multiplex PCR reactions for amplification of (*atf*A + *zap*A), (*hpm*A + *lux*S), and (*ure*C+ *uca*A). Each of the 2 genes *fla*A and *mrp*A was amplified in a single PCR reaction. The PCR reactions contained 12.5 μL 2x PCR master mix (Dream Taq™ Hot Start Green DNA Polymerase Master Mix (Thermo Fisher), 1 μL of each primer (10 pmol/μL), 3 μL of DNA extract, and PCR-grade water to a final volume of 25 μL.

The primers’ sequences, annealing temperatures, and the expected sizes of the amplicons are illustrated in Table S1, supplementary material. The thermal cycling conditions were 4 min of initial denaturation at 95 °C, 35 cycles of denaturation at 95 C for 30 s, annealing at the primers’ specific annealing temperature for 30 s, and extension for 72 °C for 1 min, followed by 10 min of final extension at 72 °C.

Amplification products were visualized by electrophoresis using 1.5% (w/v) agarose gel in TAE buffer stained with 5 μL of ethidium bromide solution (10 mg/mL). Gene-specific bands were observed in comparison with the bands of a 100-bp DNA ladder (Thermo Fisher) using a 302-nm UV transilluminator.

### Statistical analysis

Data were analyzed using IBM SPSS software package version 20.0. (Armonk, NY: IBM Corp). The significance of the obtained results was judged at the 5% alpha level. For categorical variables, the chi-square test was used to compare different groups while Fisher’s exact or Monte Carlo correction for chi-square when more than 20% of the cells have an expected count of less than 5. Correlation analysis by the Spearman rank method was done using RStudio.

## Results

The majority of *P. mirabilis* isolates were isolated from mid-stream urine samples 55 (55%) followed by wound swabs 31 (31%), and catheter-associated urinary tract infection 14 (14%).

### Results of antimicrobial susceptibility testing

**Fig. 1:**
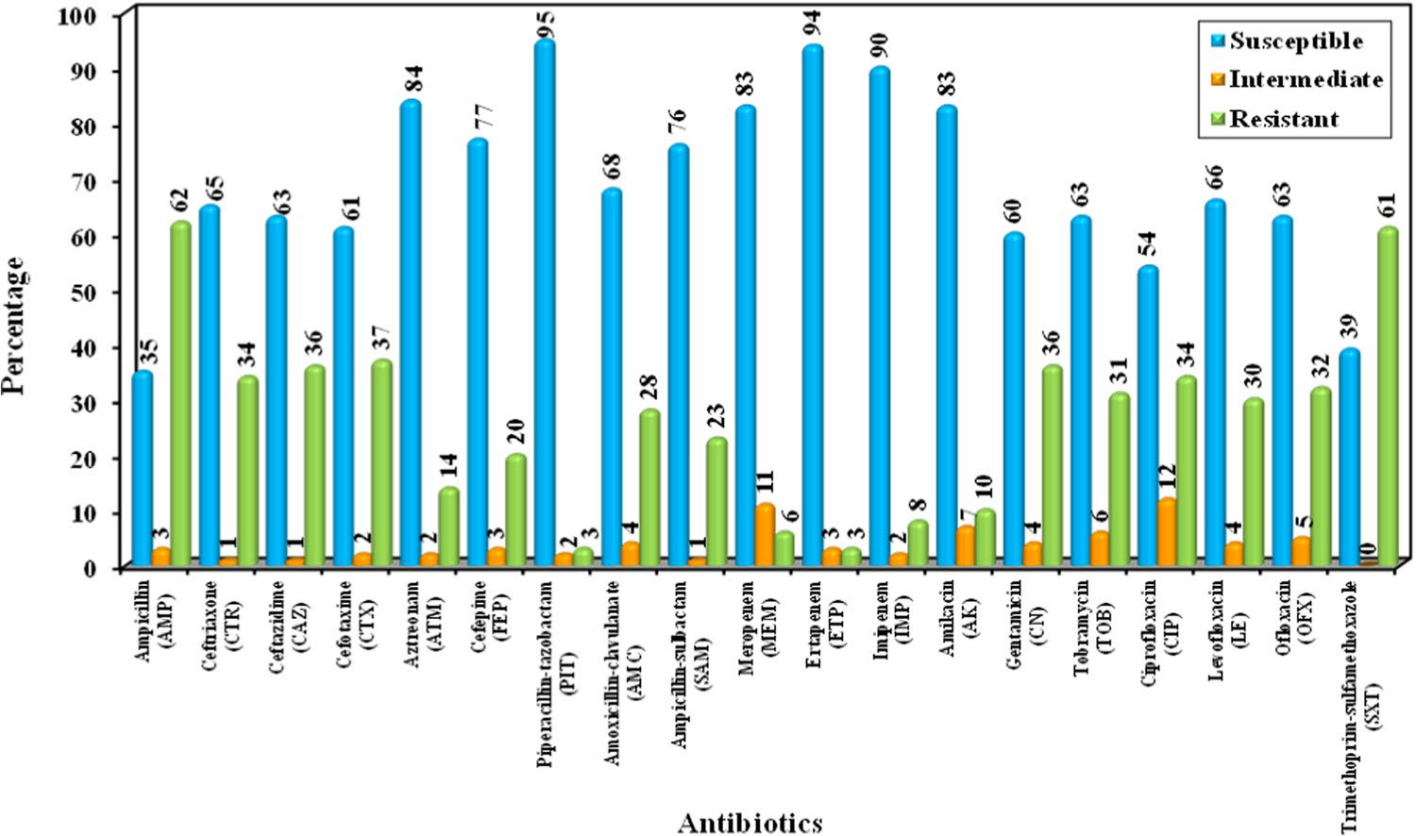
Antimicrobial susceptibility testing results of the 100 isolates of *P. mirabilis*

The highest percentage of resistance was 62% and 61% to ampicillin and trimethoprim-sulfamethoxazole, respectively, while the lowest resistance was 3%, 3%, 6%, and 8% to piperacillin-tazobactam, ertapenem, meropenem, and imipenem respectively (Fig. 1). Overall, 61% of isolates were non-MDR, 34% were MDR, and 5% were XDR.

### Phenotypic detection of virulence factors

All isolates (100%) were positive for motility, swarming, urease, and protease production. Ninety isolates (90%) were positive for hemolysin production by tube hemolysis test. Seventy- three isolates (73%) were positive for biofilm formation: including 40 isolates forming a weak biofilm, 30 forming a moderate biofilm, and only 3 isolates forming a strong biofilm.

### Genotypic detection of virulence factors

All *P. mirabilis* isolates (100%) were positive for *ure*C and *zap*A gene, encoding for urease and extracellular metalloprotease, respectively. As for the quorum sensing gene *luxS*, the flagellar gene *flaA*, and the fimbrial adhesin gene *ucaA*, they were detected in 99%, 98%, and 96% of the isolates, respectively. The hemolysin gene *hpm*A and the fimbrial genes *mrp*A and *atfA* genes were positive in 90%, 89%, and 84% of the isolates, respectively (Figs. S1–S5 supplementary material).

There was a statistically significant agreement (of 100%) between phenotypic and genotypic results in the detection of urease, protease, and hemolysin (*P* <0.001).

Among the 100 isolates, 72% carried all 8 studied virulence genes, 17% carried 7/8 of the studied virulence genes, and 8% carried 6/8 of the studied virulence genes, while 1% carried 5/8 and 2% carried 4/8 of the studied virulence genes.

There was a statistically significant association between the number of biofilm genes and biofilm formation (*P*= 0.03). The proportions did not differ between biofilm producers and non-biofilm producers except for 1 gene; *P*= 0.017, indicating that the presence of a single biofilm-related gene in the isolates (mainly *lux*S gene or *uca*A gene) was statistically significantly associated with non-biofilm production (Table 1).

**Table 1:** Distribution of biofilm-related genes and their association with biofilm formation

No. of biofilm-genes (*mrpA*, *ucaA*, *atfA*, *luxS*) (*n*= 4) *N*= 100	Biofilm producers (*n*=73)		Non-biofilm producers (*n*= 27)		*χ*^2^	*P*-value
	No.	%	No.	%		^FE^*P*= 0.03*
4 genes	57	78.1	19	70.4	0.34097	*P*^*a*^=0.5593
3 genes	15	20.5	4	14.8	0.15424	*P*^*a*^=0.6945
2 genes	1	1.4	1	3.7	0.53678	*P*^*b*^=1
1 gene	0	0.0	3	11.1	8.11*	*P*^*b*^=0.017*

*Statistically significant at *P* ≤ 0.05, ^FE^*P* was obtained from Fisher exact test, *χ*^2^, *P*^*a*^: chi-square test for goodness of fit, *χ*^2^, *P*^*b*^: chi-square test for goodness of fit with Monte Carlo simulation

### Association between virulence factors and the type of clinical infection

There was a statistically significant difference regarding biofilm formation among isolates from different types of clinical samples (*P* = 0.034). All isolates from catheterized urine samples formed biofilm (100%), followed by wound isolates (74.2%), and then mid-stream urine samples (65.5%) (Table 2).

**Table 2:** Distribution of virulence factors according to the type of clinical specimens

Virulence factors	Clinical sample types						*χ*^2^	^*MC*^*P*
	Mid-stream urine (*n*=55)		Wound swab (*n*=31)		Catheterized urine (*n*=14)			
	No.	%	No.	%	No.	%		
Hemolysis								
Positive (*n*=90)	48	87.3%	28	90.3%	14	100%	1.551	0.522
Negative (*n*=10)	7	12.7%	3	9.7%	0	0%		
Biofilm								
Positive (*n*=73)	36	65.5%	23	74.2%	14	100%	6.789*	0.034*
Weak (*n*=40)	17	30.9%	16	51.6%	7	50%	4.219	0.121
Moderate (*n*=30)	17	30.9%	7	22.6%	6	42.9%	1.936	0.380
Strong (*n*=3)	2	3.6%	0	0%	1	7.1%	2.026	0.255
Negative (*n*=27)	19	34.5%	8	25.8%	0	0%	6.789*	0.034*
Motility								
Positive (*n*=100)	55	100%	31	100%	14	100%	-	-
Negative (*n*=zero)	0	0%	0	0%	0	0%		
Swarming								
Positive (*n*=100)	55	100%	31	100%	14	100%	-	-
Negative (*n*=zero)	0	0%	0	0%	0	0%		
Urease								
Positive (*n*=100)	55	100%	31	100%	14	100%	-	-
Negative (*n*=zero)	0	0%	0	0%	0	0%		
Protease								
Positive (*n*=100)	55	100%	31	100%	14	100%	–	–
Negative (*n*=zero)	0	0%	0	0%	0	0%		

*Statistically significant at *P* ≤ 0.05; *χ*^*2*^, chi square test; *MC*, Monte Carlo

### Association between antimicrobial resistance and virulence factors in P. mirabilis isolates

There was no statistically significant difference in analyzing the distribution of virulence factors phenotypically and genotypically among isolates with different antimicrobial resistance patterns **(**Tables 3 and 4).

**Table 3:** Distribution of virulence factors among non-MDR, MDR, and XDR isolates

Virulence factors	Resistance pattern						*χ*^2^	^*MC*^*P*
	Non-MDR (*n*=61)		MDR (*n*=34)		XDR (*n*=5)			
	No.	%	No.	%	No.	%		
Motility								
Positive (*n*=100)	61	100%	34	100%	5	100%	–	–
Negative (*n*=zero)	0	0%	0	0%	0	0%		
Swarming								
Positive (*n*=100)	61	100%	34	100%	5	100%	–	–
Negative (*n*=zero)	0	0%	0	0%	0	0%		
Urease								
Positive (*n*=100)	61	100%	34	100%	5	100%	–	–
Negative (*n*=zero)	0	0%	0	0%	0	0%		
Protease								
Positive (*n*=100)	61	100%	34	100%	5	100%	–	–
Negative (*n*=zero)	0	0%	0	0%	0	0%		
Hemolysis								
Positive (*n*=90)	52	85.2%	33	97.1%	5	100%	3.258	0.179
Negative (*n*=10)	9	14.8%	1	2.9%	0	0%		
Biofilm								
Positive	43	70.5%	25	73.5%	5	100%	5.771	0.440
Weak (*n*=40)	21	34.4%	17	50.0%	2	40%		
Moderate (*n*=30)	20	32.8%	7	20.6%	3	60%		
Strong (*n*=3)	2	3.3%	1	2.9%	0	0%		
Negative	18	29.5%	9	26.5%	0	0%		

*χ*^*2*^, chi square test; *MC*, Monte Carlo

**Table 4:** Distribution of virulence genes among non-MDR, MDR, and XDR isolates

Virulence genes	Resistance pattern						*χ*^2^	^*MC*^*P*
	Non-MDR (*n*=61)		MDR (*n*=34)		XDR (*n*=5)			
	No.	%	No.	%	No.	%		
*flaA*								
Positive (*n*=98)	60	98.4%	33	97.1%	5	100%	1.287	1.000
Negative (*n*=2)	1	1.6%	1	2.9%	0	0%		
*ureC*								
Positive (*n*=100)	61	100%	34	100%	5	100%	–	–
Negative (*n*=zero)	0	0%	0	0%	0	0%		
*zapA*								
Positive (*n*=100)	61	100%	34	100%	5	100%	–	–
Negative (*n*=zero)	0	0%	0	0%	0	0%		
*hpmA*								
Positive (*n*=90)	52	85.2%	33	97.1%	5	100%	3.258	0.179
Negative (*n*=10)	9	14.8%	1	2.9%	0	0%		
*ucaA*								
Positive (*n*=96)	58	95.1%	33	97.1%	5	100%	–	–
Negative (*n*=4)	3	4.9%	1	2.9%	0	0%		
*mrpA*								
Positive (*n*=89)	55	90.2%	29	85.3%	5	100%	0.762	0.736
Negative (*n*=11)	6	9.8%	5	14.7%	0	0%		
*atfA*								
Positive (*n*=84)	49	80.3%	30	88.2%	5	100%	1.322	0.502
Negative (*n*=16)	12	19.7%	4	11.8%	0	0%		
*luxS*								
Positive (*n*=99)	61	100%	33	97.1%	5	100%	3.071	0.396
Negative (*n*=1)	0	0%	1	2.9%	0	0%		

*χ*^*2*^, chi square test; *MC*, Monte Carlo

Only 70.5% of the non-MDR and 73.5% of MDR *P. mirabilis* isolates were biofilm producers, while all 5 XDR isolates produced biofilm. Most of the isolates formed weak to moderate biofilm, regardless of their resistance patterns. The 73 biofilm-forming isolates included 43 non-MDR isolates, 25 MDR, and 5 XDR. Among the 43 non-MDR isolates, 21 formed weak biofilm, 20 formed moderate, and 2 formed strong biofilm. As for the 25 MDR biofilm-forming isolates, 17 formed weak biofilm, 7 formed moderate biofilm, and 2 formed strong biofilm. Two of the 5 XDR isolates formed weak biofilm, while 3 formed moderate biofilm. Among the 27 isolates that were unable to form a biofilm, 18 were non-MDR and 9 were MDR (Table 3).

The quorum sensing gene *lux*S was detected in all the non-MDR *P. mirabilis* isolates, followed by the flagellar gene the *fla*A which was detected in 98.4% of these isolates. The hemolysin gene *hpm*A was positive in 85.2%. The prevalence of fimbrial adhesin genes (*uca*A, *mrp*A, and *atf*A) was 95.1%, 90.2%, and 80.3%, respectively (Table 4).

As for the MDR isolates, the quorum sensing gene, *lux*S, was positive in 97.1% of the isolates. Both the flagellar gene, *fla*A, and hemolysin gene, *hpm*A, were positive in 97.1% of them. The prevalence of fimbrial adhesin genes (*uca*A, *atf*A, and *mrp*A) was 97.1%, 88.2%, and 85.3%, respectively. All the XDR isolates were positive for all the aforementioned genes.

### Correlation between different virulence genes, virulence factors, and resistance patterns among all *P. mirabilis* isolates

**Fig. 2:**
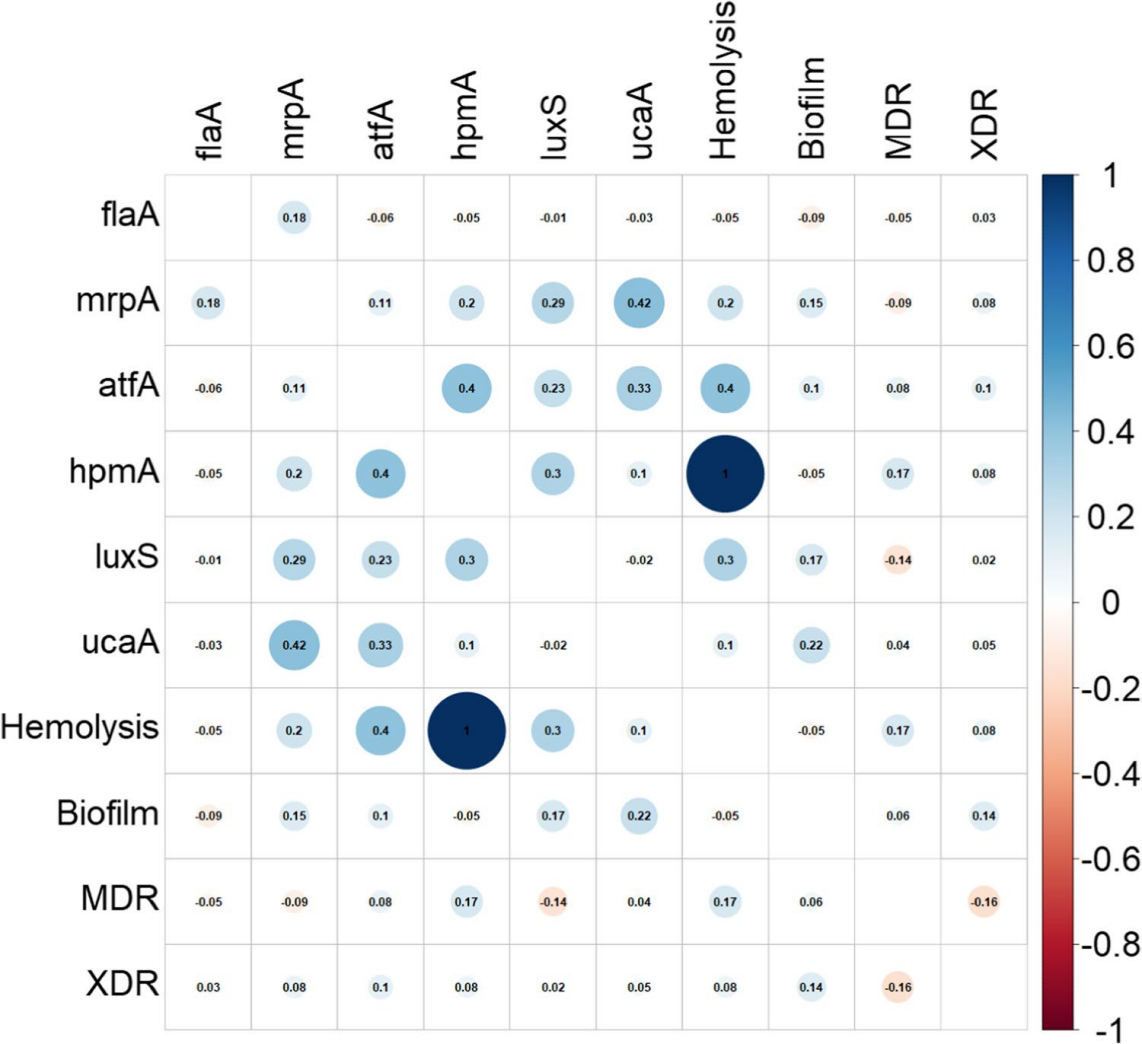
Heat map of correlation analysis by Spearman rank method between different virulence genes, virulence factors, and resistance patterns among all *P. mirabilis* isolates (*n*= 100). Blue color indicates a positive correlation while red color indicates a negative correlation. This figure was produced by the Corrplot package in RStudio. A value of +1 or *−*1 indicates a perfect correlation, 0.8 to 0.9 (*−*0.8 to *−*0.9) indicates a very strong correlation, 0.6 to 0.7 (*−*0.6 to *−*0.7) indicates a moderate correlation, and 0.3 to 0.5 (*−*0.3 to *−*0.5) indicates a fair correlation

There was a significant positive correlation between the following pairs of virulence genes: *mrp*A and *uca*A (rho= 0.42, *P*= 0.0007), *atf*A and *hpm*A (rho=0.4, *P*= 0.002), *atf*A and *uca*A (rho= 0.33, *P*= 0.03). A perfect positive significant correlation between *hpm*A virulence gene and the hemolysis virulence factor was observed (rho= 1, *P*<0.000001). Moreover, a statistically significant positive correlation was noticed between the *atf*A virulence gene and hemolysis (rho= 0.4, *P*=0.002). Despite the fact that the rho coefficients between virulence genes are small ranging 0.33 to 0.42 indicating a fair positive correlation, the correlation is statistically significant and cannot be ignored (Fig. 2). No correlation was detected between virulence factors and antibiotic resistance patterns (MDR and XDR).

## Discussion

Multidrug resistance in *P. mirabilis* is increasingly observed. The high level of intrinsic resistance and the high virulence of this bacterial species further augment its impact on public health. Multidrug-resistant *P. mirabilis* has been previously isolated from a variety of human clinical specimens and food animals [30]. In this study, we determined the antibiotic resistance profiles of 100 clinical isolates of *P. mirabilis*, collected from urine samples and wound infection*.* In addition, we examined the isolates phenotypically and genotypically for the presence of some important virulence determinants including motility, swarming, urease and protease production, biofilm formation, and quorum sensing. We aimed to explore any potential correlations between virulence determinants and multidrug resistance among clinical isolates of *P. mirabilis*.

Multidrug resistance in *P. mirabilis* has been reported to be gained by means of horizontal gene transfer from other bacterial species such as *K. pneumoniae*, with the eventual formation of a hybrid (mosaic) plasmid that carries resistance and virulence genes from both species [31]. In our study, we detected multidrug resistance and extensive drug resistance in 34% and 5% of our isolates, respectively. This percentage is less than that reported in Israel in 2010, where the authors reported the isolation of MDR *P. mirabilis* from 50% of their patients who were all hospitalized with UTI [32]. A more recent study in India also revealed a much higher prevalence of MDR (85%) among UTI isolates of *P. mirabilis* [33]. Lower prevalence, however, was reported in Europe and Taiwan [34, 35].

Four out of the six studied virulence factors were invariably detected phenotypically among all our isolates of *P. mirabilis*: motility, swarming, urease, and protease production. However, at the molecular level, only *ure*C and *zap*A genes were amplified in all isolates. The flagellar gene *fla*A was amplified in 98% of the isolates.

With regard to our two motile isolates with non-amplified *flaA* gene, this result could be explained by the recombination of *fla*A and *fla*B with the formation of *fla*AB hybrids that have different nucleotide sequence that is not amplifiable by the *fla*A primers used in this study [36]. Another possible explanation is that the swarming was controlled by other genes such as *fli*L gene [37].

Similar results for the detection of virulence factors in urine isolates of *P. mirabilis* were also encountered by Filipiak et al. who found that all isolates of *P. mirabilis* did produce two of the studied virulence determinants phenotypically: swarming and urease production. They also reported the amplification of *ure*C and *zap*A genes in all isolates [38]. Likewise, Abd Al-Mayahi and Al-Dulaimi et al. detected *fla*A gene in all the swarming isolates [39, 40]. On the contrary, Ali et al. reported a lower percentage of *flaA* gene amplification (86.66%) among swarming isolates [25]. On the other hand, Al-Dulaimi et al. detected *ure*C gene in only (85.7%) of their urease-producing isolates [40]. In addition, Alsherees et al. detected *zap*A gene in only (39.28%) of their isolates [24].

Hemolysis was detected phenotypically and genotypically (*hpm*A amplified) in 90% of our isolates. On the other hand, Filipiak et al. detected *hpm*A gene in all isolates although hemolytic activity was phenotypically observed in 84% of their isolates, with only 16% showing typical appearance of beta hemolysis [38]. Similarly, Mirzaei et al. reported that all their *P. mirabilis* isolates produced hemolysin and had *hpm*A gene [33]. On the contrary, Jaber et al. detected *hpm*A gene in only 50% of their isolates [41].

Seventy-three percent of our isolates were able to form biofilm on microtiter plates, which was mostly of weak (40%) to moderate (30%) intensity. At the molecular level, biofilm-related genes, *luxS* quorum sensing gene, and the fimbrial genes: *uca*A, *mrp*A, and *atf*A were amplified in of 99%, 96%, 89%, and 84% of the isolates respectively.

The sole presence of one biofilm-related gene in the isolates (mainly *luxS* gene or *ucaA* gene) was statistically associated with non-biofilm production (*P*=0.017). A statistically significant fair positive correlation was detected between some of the biofilm genes: *mrp*A and *uca*A (*P*= 0.0007), as well as *atf*A and *uca*A (*P*= 0.03). The fimbrial gene *atf*A was also found to be of a fair positive correlation with hemolysis (*P*=0.002) and *hpmA* gene (*P*= 0.002).

The relation between *lux*S gene and biofilm production was previously reported by Abd Albagar et al. [42]. However, other studies suggested that MR/P and ATF fimbriae rather than UCA fimbriae have a major role in biofilm formation [43, 44]. Based on the results of the current study, no significant correlation was detected between *lux*S gene and *uca*A genes and they were not found to be significantly correlated with biofilm production.

The association between the high abundance of biofilm-related genes among the isolates and their ability to form biofilm phenotypically was also observed by Filipiak et al. who reported that the biofilm-genes *uca*A and *mrp*A were amplified in all of their *P. mirabilis* isolates and 96% of them were able to form biofilm on polyurethane [38]. Several studies also reported a high abundance of biofilm genes among clinical isolates of *P. mirabilis*. For instance, Hussein et al. detected *lux*S and atfA genes in 100% and 98.4% of their isolates, respectively [45]. In addition, Kamel et al. detected *mrp*A gene in all tested isolates [46]. On the other hand, Abbas et al. reported that only 47% of their isolates carried *lux*S gene, while 35% carried *mrp*A gene [47]. Sun et al. reported a lower percentage of *uca*A (33%) and *atfA* (64.77%) genes among their isolates of *P. mirabilis* [43].

Catheterized urine isolates are more liable to form biofilm than mid-stream urine isolates, since catheter surfaces facilitate adherence of bacteria without clearance, in the absence of host defense mechanisms which occur in the bladder upon binding of bacteria to epithelia, and also in the absence of the flushing mechanism of urine which also has a role [6, 48]. From this work, we observed that all the isolates from catheterized urine samples were able to form a biofilm, followed by wound swab isolates (74.2%). A statistically significant difference (*P*=0.034) was observed regarding the distribution of biofilm-positive isolates among different types of clinical samples.

Similarly, Jacobsen et al. proved that all *P. mirabilis* isolates from urinary catheters formed a biofilm [8]. Also, Hola et al. reported that catheterized urine *P. mirabilis* isolates had a higher ability to form biofilm than those isolated from feces as a control group [49]. Another similar study reported by Abdallah et al. found that *P. mirabilis* isolates from catheterized patients had a higher percentage (43.3%) of biofilm-forming ability than those of non-catheterized samples (30%) but did not reach the level of significance [50]. On the contrary, Kwiecinska-Piróg et al. reported no difference in biofilm formation ability between catheterized and non-catheterized urine isolates [22]**.**

Our results revealed that the existence of virulence factors, including the ability to form a biofilm, is not correlated with the antimicrobial resistance profile of *P. mirabilis* clinical isolates. All of our isolates were positive to 4/6 of the studied virulence factors, regardless of being MDR or non-MDR. Nevertheless, it was observed that all the XDR isolates were positive to all the six studied virulence factors phenotypically and at the molecular level.

On the other hand, a negative correlation between virulence and multidrug resistance was reported by Rodulfo et al. who found that non-MDR *P. mirabilis* possessed a higher number of virulence factors compared to MDR, yet this was statistically significant only with 2 virulence factors that are swarming and twitching motility [51].

The *hpm*A hemolysin of *P. mirabilis* is accountable for pore formation in host cells with subsequent tissue damage [52]. This hemolysin was not detected phenotypically and genotypically in 10 of our 100 isolates, among which 9 were non-MDR. Likewise, Mishu et al. reported that among their 44 clinical isolates of *P. mirabilis*, 3 isolates were negative for *hpm*A, among which 2 were non-MDR and 1 was MDR [53].

According to Sun et al., the formation of moderate-intensity biofilm is correlated with increased virulence [43]. This agrees with our findings, as most of our isolates that were positive for biofilm formation, which was of moderate to weak intensity, were also positive for most of the studied virulence determinants.

We did not find any significant association between biofilm formation and multidrug resistance. Our results showed that biofilm formation was slightly higher among non-MDR isolates (58.9%). At the same time, most of the non-biofilm-forming isolates were also non-MDR (66.7%). However, all the XDR isolates were biofilm producers. Moreover, most of the MDR isolates (73.5%) were also biofilm producers. This high biofilm-forming ability of XDR and MDR isolates of *P. mirabilis* complicates infection and renders treatment more difficult.

Similar findings were reported by Rodulfo et al., who found that biofilm formation was slightly higher in the non-MDR isolates of *P. mirabilis* (84.8%), compared to 76.1% in the MDR group [51]. Meanwhile, Ghaima et al. and Sun et al. reported that biofilm-forming isolates of *P. mirabilis* significantly displayed more resistance to antimicrobial agents compared with non-biofilm-forming isolates [43, 54]. Also, Filipiak et al. reported that strong biofilm formation is correlated with multidrug resistance, and they attributed this to the blockade of antimicrobial penetration by the extracellular matrix of biofilm [38].

## Conclusion

From this work, we concluded that *P. mirabilis* isolates collected from catheterized-urine samples are associated with a high ability of biofilm formation. A significant positive correlation was detected between some pairs of virulence genes in *P. mirabilis*: *mrp*A and *uca*A, in addition to *atf*A and *uca*A, as well as *atf*A and *hpm*A. The ability of biofilm formation and the high abundance of virulence factors were not found to be correlated with multidrug resistance. The non-MDR isolates of *P. mirabilis* have a large repository of virulence factors with no statistically significant difference from MDR isolates. Most of the MDR and all XDR isolates were biofilm producers, which represents a serious challenge in the management of infection by these isolates.

This study reveals that most clinical isolates of *P. mirabilis*, regardless of their resistance pattern, are fully equipped with a large number of virulence factors, the co-existence of many of which is significantly correlated.

## Supplementary information

ESM 1 (DOCX 1649 kb)
