# Supplementary material for: A correlation study between virulence factors and multidrug resistance among clinical isolates of Proteus mirabilis
Source: Braz J Microbiol. 2023 Aug 3;54(3):1387–97. doi: 10.1007/s42770-023-01080-5 (PMC10484824; doi:10.1007/s42770-023-01080-5)
Supplement: Supplementary file 1 — (DOCX 1649 kb) [file 42770_2023_1080_MOESM1_ESM.docx]

**Brazilian Journal of Microbiology**

**A Correlation study between virulence factors and multidrug resistance among clinical isolates of *Proteus mirabilis***

Mai Elhoshi ^1^, Eglal El-Sherbiny ^1^, Amel Elsheredy ^1^ , Aliaa Gamaleldin Aboulela ^1^ *

^1^ Medical Research Institute, Alexandria University, Department of Microbiology, Alexandria, Egypt

* Corresponding author: Aliaa Gamaleldin Aboulela (PhD), aliaagamaleldin@alexu.edu.eg

**Table S1:** **Primers for the detection of target genes**

| **Gene** | **Nucleotide sequence of primers** | **Amplicon size (bp)** | **Annealing temperature** | **Reference** |
| --- | --- | --- | --- | --- |
| *zap*A  (Encoding extracellular metalloprotease) | F: 5' - ACCGCAGGAAAACATATAGCCC −3' | 540 | 58°C | [23] |
|  | R: 5'- GCGACTATCTTCCGCATAATCA-3' |  |  |  |
| *fla*A  (Encoding flagellae) | F: 5'-AGGATAAATGGCCACATTG-3' | 417 | 50°C | [24] |
|  | R: 5'-CGGCATTGTTAATCGCTTTT-3' |  |  |  |
| *ure*C  (Encoding urease enzyme large subunit) | F: 5'-CCGGAACAGAAGTTGTCGCTGGA-3' | 533 | 60°C | [25] |
|  | R: 5'- GGGCTCTCCTACCGACTTGATC-3' |  |  |  |
| *mrp*A  (Encoding Mannose-resistant Proteus-like fimbria) | F:5'-ATTTCAGGAAACAAAAGATG-3' | 565 | 50°C | [26] |
|  | R: 5'- TTCTTACTGATAAGACATTG-3' |  |  |  |
| *atf*A  (Encoding ambient-temperature fimbriae) | F: 5'-CATAATTTCTAGACCTGCCCTAGCA-3' | 382 | 58°C | [27] |
|  | R: 5'-CTGCTTGGATCCGTAATTTTTAACG-3' |  |  |  |
| *uca*A  (Encoding uroepithelial cell adhesin fimbriae) | F: 5'-AACCAGTTCCGCGTTGGCCTGG-3' | 690 | 60°C | [28] |
|  | R: 5'-CGGAACGGCCTGACGTTGCAT-3' |  |  |  |
| *hpm*A  (Encoding hemolysin) | F: 5'-TGGTATCGATGTTGGCGTTA-3' | 717 | 54°C | [24] |
|  | R: 5'- GTGGTGCCCACTTTCAGATT-3' |  |  |  |
| *lux*S  (Involved in quorum sensing) | F: 5'-GTATGTCTGCACCTGCGGTA-3' | 464 | 54°C | [15] |
|  | R: 5'- TTTGAGTTTGTCTTCTGGTAGTGC-3' |  |  |  |

F: forward, R: reverse


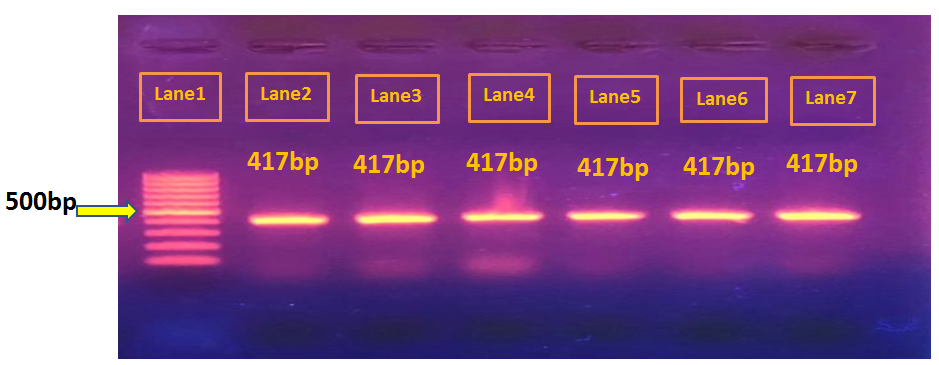


**Fig. S1: Ethidium bromide-stained agarose gel electrophoresis for the detection of *fla*A amplicon*.* Lane 1 contains a 100 bp DNA ladder. Lanes 2, 3, 4, 5, 6, and 7 show positive bands for *fla*A amplicon at 417 bp.**


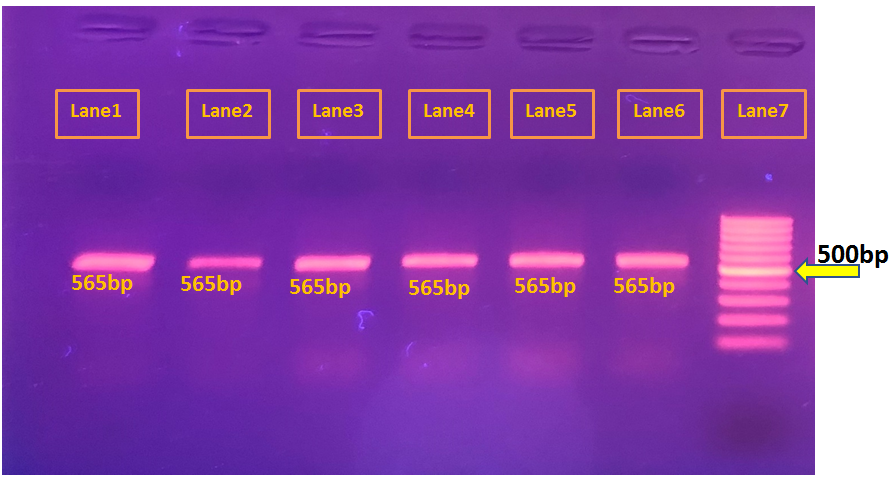


**Fig. S2: Ethidium bromide-stained agarose gel electrophoresis for the detection of mrpA amplicon. Lane 7 contains a 100 bp DNA ladder. Lanes 1, 2, 3, 4, 5, and 6 show positive bands for mrpA amplicon at 565 bp.**


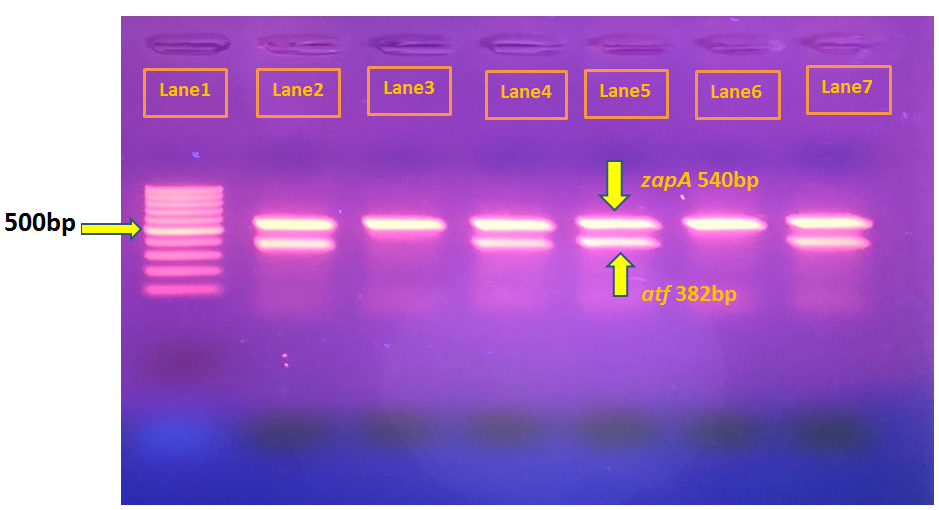


**Fig. S3: Ethidium bromide-stained agarose gel electrophoresis after multiplex PCR for detection of *zap*A and *atf*A amplicons*.* Lane 1 contains a 100 bp DNA ladder. Lanes 2, 3, 4, 5, 6, and 7 show positive bands for *zap*A amplicon at 540bp. Lanes 2, 4,5, and 7 show positive bands for *atf*A amplicon at 382 bp.**


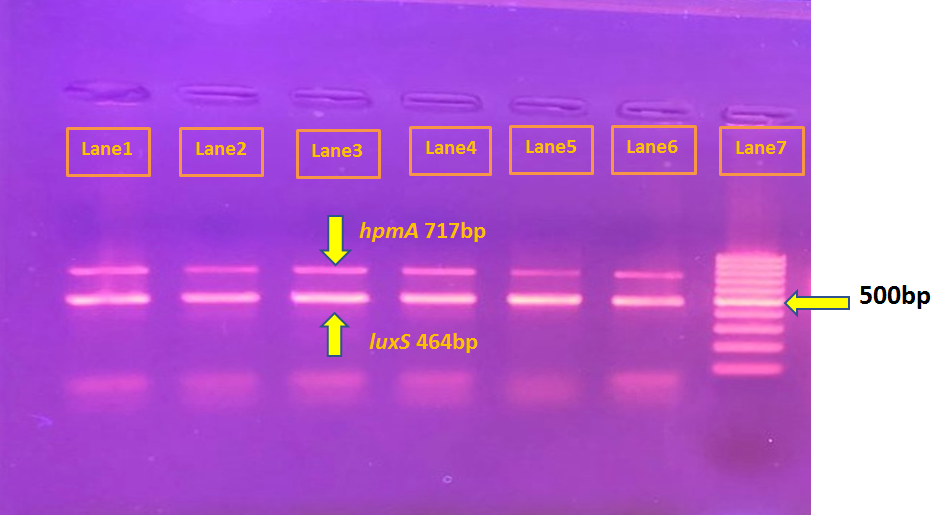


**Fig. S4: 2 Ethidium bromide-stained agarose gel electrophoresis after multiplex PCR for detection of *hpm*A and *lux*S amplicons*.* Lane 7 contains a 100 bp DNA ladder. Lanes 1, 2, 3, 4, 5, and 6 show positive bands for *hpm*A amplicon at 717bp. Lanes1, 2, 3, 4, 5, and 6 show positive bands for *lux*S amplicon at 464 bp.**


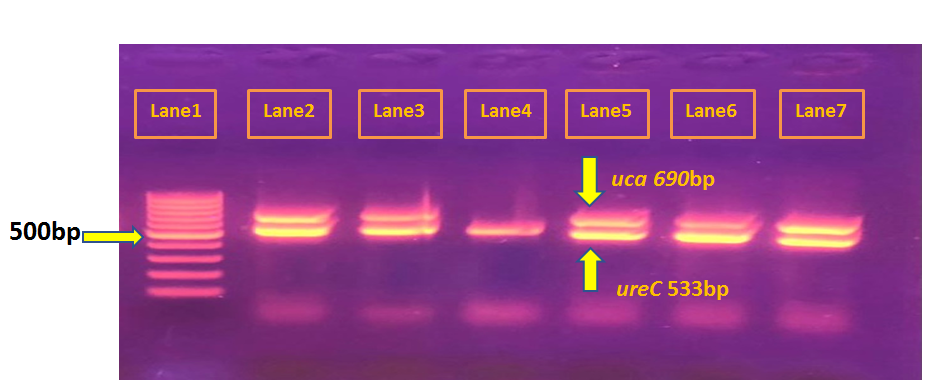


**Figure S5: Ethidium bromide-stained agarose gel electrophoresis after multiplex PCR for detection of** ***uca*A *and ure*C amplicons*.* Lane 1 contains a 100 bp DNA ladder. Lanes 2, 3, 4, 5, 6 and 7 show positive bands for *ure*C amplicon at 533 bp. Lanes 2, 3,5, 6 and 7 show positive bands for *uca*A amplicon at 690 bp.**
